# Nonpharmacological measures to prevent allergic symptoms in pollen allergy: A critical review 

**DOI:** 10.5414/ALX02294E

**Published:** 2021-12-01

**Authors:** Karl-Christian Bergmann, Markus Berger, Ludger Klimek, Oliver Pfaar, Barbora Werchan, Matthias Werchan, Torsten Zuberbier

**Affiliations:** 1 Charité, Universitätsmedizin Berlin, Clinic for Dermatology, Venereology and Allergy, Berlin, Germany,; 2 Institut für Pathophysiologie und Allergieforschung, Zentrum für Pathophysiologie, Infektiologie und Immunologie, Medizinische Universität Wien, Vienna, Austria,; 3 Center for Rhinology and Allergology, Wiesbaden,; 4 Department of Otorhinolaryngology, Head and Neck Surgery, Section of Rhinology and Allergy, University Hospital Marburg, Philipps-Universität Marburg, Marburg,; 5 German Pollen Information Service Foundation (PID), Berlin, Germany

**Keywords:** pollen, allergic rhinitis, avoidance, prevention, non-drug methods

## Abstract

Allergic rhinoconjunctivitis (hay fever) is the most common chronic disease in all industrialized nations. Therapy consists essentially in the use of anti-allergic and anti-inflammatory drugs, which mostly show a good and quick effect. With allergen-specific immunotherapy, there is also a causal possibility of tolerance induction. There is currently a considerable undersupply, as those affected trivialize the symptoms and often have concerns about long-term drug therapy. There is also great interest in using non-medicinal measures to prevent and/or relieve allergic symptoms on the assumption that these are free from side effects. In this publication, we present non-drug methods for which clinical studies are available in the literature. The methods have varying degrees of effectiveness. An evidence-based comparative assessment between the methods is not possible. There are also hardly any studies in comparison to standard drug therapy. A large number of the interventions consist of allergen reduction, e.g., with air filters, or cleaning of the mucous membranes with nasal irrigation, etc., none of which should be seen as a substitute but as a supplement to drug therapy.

## Introduction 

Without doubt, allergic rhinoconjunctivitis (ARC) due to pollen is a widespread disease and the most common allergic disease in Europe. Based on surveys of physician-diagnosed diseases, the lifetime prevalence of hay fever is 14.8% for adults in Germany and 10.7% for children and adolescents aged 0 – 17 years [58]. Thus, ~ 10.1 million adults and 1.4 million children and adolescents suffer from seasonal symptoms during the pollen season; in addition, an unknown number of individuals suffer from pollen-triggered bronchial asthma and pollen-triggered oral allergy syndrome. 

The effects of pollen allergy mean a significant reduction in the quality of life for individuals as a result of organ-specific and general (“hay fever”) symptoms. For children and adolescents, hay fever leads to impaired learning ability [61] and for society as a whole to a loss of gross national product. Thus, in addition to personal limitations, this common disease is costly to society as a whole [16, 68]. 

Drug therapy for ARC is the standard of care in the management of the disease [40]; it is successful, but not without side effects. Impaired attention and increased need for sleep are most common with the use of anti-allergic medication. 

Allergen immunotherapy (AIT) is just as successful, but the only disease-modifying therapy option that influences the underlying pathomechanism of the disease [52,53,54]; unfortunately, AIT, it is not performed to the extent that would be desirable. The barriers to using allergen-specific immunotherapy are many, for example, the long, 3-year duration and the difficulty in adherence to constant intake. 

Many patients, for various reasons, wish to be completely or partially helped by non-medicinal means and also resort to untested and/or meaningless methods. One example is the bioresonance method, but things have quieted about it lately. 

Here an attempt is made to give an overview of the non-medicinal measures recommended in the literature for the prevention or alleviation of symptoms in ARC. Those methods are mentioned that showed at least partial efficacy in clinical trials and were published in scientific journals after peer review (Table 1). 

## Acupuncture 

Acupuncture is cultivated especially in Chinese medicine in different methodological variants and has also been applied in persons with ARC. 

Treatment over 8 weeks with 12 acupunctures demonstrated a significant improvement in disease-specific quality of life and reduced use of anti-histamines compared to sham acupuncture in over 400 participants [15]; however, the effect was no longer detectable 1 year later. A small study of 30 participants found significant effects with 12 treatments within 4 weeks in symptom relief as well, but concomitant medication remained high [64]. 

In another study by the authors [65], rhinitis symptoms (nasal itching, sneezing) were found reduced in 175 participants after 4 weeks of treatment, again with 12 acupuncture treatments. After another 4 weeks without therapy, the symptoms remained statistically significantly reduced compared to sham acupuncture. 

A meta-analysis in 2015 [25], which evaluated 13 publications, came to an overall positive conclusion; 1,126 treated subjects with ARC were compared with 1,239 subjects from control groups. The subjects treated with acupuncture showed a significant reduction in nasal symptoms, medication use, and even a reduction in total blood IgE. Quality of life, as measured by the Rhinitis Quality of Life Questionnaire (RQLQ) and the 36-item Short-Form (SF-36), also showed improvement in the sum of these publications in meta-analysis side-effects do not appear to occur with acupuncture. 

The most recent meta-analysis from 2020 evaluated the results of 39 published studies with 3,433 participants and also concluded that all acupuncture methods studied had significantly better outcomes than sham acupuncture in terms of nasal symptoms and quality of life [66]. The method of moxibustion (heating specific points of the body) produced the best results, but the combination of manual acupuncture in combination with drug therapy was also successful. 

For individuals with ARC who do not respond adequately to standard drug therapy or experience intolerable side effects, acupuncture may be of value. Presumably, the effect will depend greatly on the experience of the acupuncturist and possibly on the willingness of the patient to engage in the methodology. 

## Car filters 

The filters installed in all cars today effectively retain particulate matter from ~ 0.7 to 74 µm, regardless of its sources (plant, animal, metal, road debris). Thus, even whole pollen and fragments of pollen are regularly excluded from entering the car with the windows closed [33] and should protect drivers suffering from ARC. A clinical study demonstrating the beneficial effect of car filters during car travel does not appear to have been published to date. On the other hand, there are studies that allergies are responsible for up to 7% of traffic accidents, including reflex eyelid closure during sneezing [19]. 

However – even the best filters in cars age, and it has been demonstrated that the filtering effect of small particles from outdoor air (PM_2.5_) diminishes [62]. Without being able to refer to published data, it can be recommended to pollen allergy sufferers to change the filter ~ every 2 years. 

## Plants in private and public spaces 

The types of plants in one’s garden, front yard, or in public spaces can influence exposure to pollen allergens. It certainly makes a difference whether a birch tree is right outside your bedroom window or a few hundred feet away. 

Suggestions for protecting pollen allergy sufferers by selecting plants in new plantings in cities have been made in Germany [11]. Recommendations for allergy-friendly planting in cities have also been published for the Mediterranean region [17]. 

Although it cannot be assumed that avoiding the planting of, for example, birch trees in a city will completely prevent the occurrence of birch pollen, their absence will contribute to a reduced amount of pollen in the air. Similarly, by inhibiting the spread of allergenic species in the city, for example ragweed and tree of heaven, one can at least reduce new sensitizations. 

In general, it can be recommended to prefer insect-pollinated species over wind-pollinated species in plantings, if possible, and also to achieve a diversity of species [32]. 

## Endonasal phototherapy 

Since endonasal phototherapy is effective in the treatment of atopic dermatitis, inducing immunomodulatory effects, phototherapy has also been tested in ARC. 

When a mixture of UVA, UVB, and visible light was applied to the nose of 62 adults (31 treated and 31 controls) in a randomized, single-blind study, there were significant improvements in the total nasal symptom score (TNSS), global severity score (GSS), and RQLQ [3]. 

In a first meta-analysis from 2015 [18], the authors confirmed significant changes with phototherapy compared to baseline: decreased nasal symptoms, improved quality of life, and significant changes in endoscopy. The improvements also involved comparisons to sham therapies and to antihistamine therapy. 

A later meta-analysis from 2017 considering 5 randomized and 7 clinical trials (mostly in China) also concluded that local phototherapy leads to significant improvements in TNSS and RQLQ without significant side effects [60]. 

However, based on knowledge from dermatology and general considerations of possible epithelial damage to mucous membranes, it must be pointed out that local application of UV light is not without risk, especially on a mucosal surface where such application is nonphysiologic. Therefore, this method cannot be recommended. 

## Hypnosis and self-hypnosis 

The complex interactions between the central nervous system, the mind, and the immune system can used as the basis for attempts to reduce the severity of allergic symptoms through hypnosis and self-hypnosis. Studies have existed for decades, but controlled trials are difficult to conduct by design and therefore rare [63]. 

A randomized parallel-group comparison of 79 adults with ARC due to birch or grass pollen over 2 years documented the effect of learned self-hypnosis on runny nose, diary entries, a VAS and use of anti-allergic medications. There were improvements in the VAS when comparing the beginning and end of the pollen season. Under self-hypnosis, nasal symptoms were fewer at the end of the 2nd season, and medication use was lower in the 1st year. Nasal provocation showed a small, but not significant, influence of self-hypnosis [42]. 

Self-hypnosis was easily learned by allergic patients and resulted in a clear, albeit statistically small, effect on symptoms of ARC and the amount of medication used [13]. The effect of hypnosis on nasal mucosal swelling was also detectable in the provocation test, but these data are not consistent [13]. 

## Artificial tears and eye compresses 

Artificial tears and cooling eye compresses are rarely used in allergic conjunctivitis. In an exposure chamber study, both options were tested in 18 subjects with ARC due to grass pollen and found to be useful. Both methods were able to significantly reduce acute eye symptoms, with artificial tears being more effective than eye compresses [12]. 

## Air purifiers 

Exposure to pollen is usually perceived as an outdoor-only exposure, but studies have demonstrated the presence of birch and grass pollen in homes, schools, and businesses as well [23, 34, 67]. Indoor pollen can then lead to persistent symptoms even after or outside the pollen season, and it is difficult to identify their cause [24]. This is where air purifiers can be applied. 

**Figure 1:**
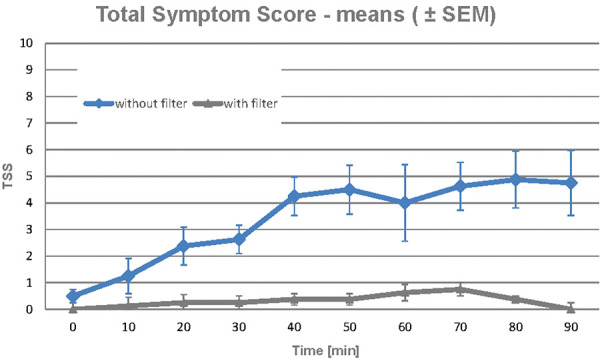
Evolution of total symptom score in 4 subjects with allergic rhinoconjunctivitis due to grass pollen during exposure to grass pollen (4,000 pollen/m^3^) for 90 minutes with and without a filter in an air purifier. From [10].

The effect of an air purifier (Philips AC4012 Air Purifier , Philips, Amsterdam, Netherlands), on intensive exposure to 4,000 grass pollen/m^3^ of air for 90 minutes was documented in a highly standardized exposure chamber. The purifier resulted in a significant reduction in nasal, conjunctival, and bronchial symptoms compared to exposure to the same amount of pollen and active air purifier with the filter removed; total symptom score and total nasal score were significantly reduced with filter use (Figure 1) [10]. 

Fixed installation of an air purifier in the bedroom of subjects with hay fever showed that air filtering at night can significantly reduce symptoms in patients with hay fever; at the same time, morning peak flow was significantly higher than in control subjects. If the subjects had perennial allergy at the same time, the effects could not be shown. The authors recommended the installation of an air filtration system for subjects with ARC. The symptom reduction shown was relatively small; however, the overall symptom severity of subjects in the active and placebo groups was also not high [14]. 

A double-blind placebo-controlled (DBPC) study (with and without built-in filters) tested the effect of an air purifier in the bedroom in 90 subjects with AR due to mugwort pollen [43]. Primary outcome parameter was a visual analogue scale with nasal symptoms significantly reduced under “real filtration”. 

A more recent study [50], also DBPC, reviewed the effect of air purifiers with HEPA filters in the bedroom and living room over 6 weeks in relation to nasal symptoms and medication – but in subjects with ARC triggered by mites. Success was multifaceted: fewer symptoms, less medication, better quality of life, and reduced concentrations of PM_2.5_ and PM_10_. Mites do not behave like pollen, but the message regarding airborne allergen particles is clear: they are significantly reduced and therefore lead to fewer symptoms. 

According to the present knowledge, the exposure of indoor spaces to infiltrated pollen and its reduction by air filters is a hitherto underestimated possibility of prevention also of pollen allergy, which can be effective and should be investigated more intensively [9]. 

## Mouth-nose masks (pollen masks) 

Mouth-nose masks should be suitable not only to reduce airborne pollutants and corona viruses during inhalation, but also particulate airborne allergens. There are a few examples of this. 

An early Japanese paper tested the effect of face masks (nose and mouth) in combination with wearing sunglasses in individuals with pollen allergy to Japanese cedar (*Cryptomeria japonica*) [31]. The number of pollen in the nasal cavity and on the eye was described as significantly reduced by the masks and glasses, but was dependent on wind. In stronger winds, the protective effect of the mask and goggles combination was lower. 

A questionnaire survey of nurses in an Israeli hospital found evidence among those with AR that wearing a medical mask and/or N95 mask (equivalent to FFP2 mask) while on duty in the hospital would reduce their allergic symptoms overall [21]. It can be assumed that pollen, mold spores, animal dander, and dust mite allergens are present only in low concentrations in the clinic – but an effect apparently existed. 

To evaluate a protective effect of mouth-nose masks, 14 adults with confirmed ARC due to grass pollen for at least 2 years outside the grass pollen season were exposed to a high dose of grass pollen for 2 hours in a standardized exposure chamber. They wore either no mask, a medical mask, or an FFP2 mask. 

**Figure 2:**
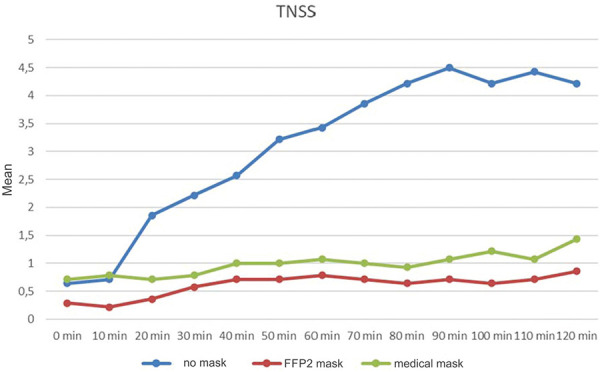
Evolution of the total nasal symptom score over 120 minutes during provocation with grass pollen without mask (blue), with FFP2 mask (red) and medical mask (green). From Bergmann et al. 2021 [9].

It was documented that wearing the masks led to a significant avoidance of nasal and also conjunctival symptoms (Figure 2). The general well-being (visual analog scale) clearly increases by wearing the masks when exposed to pollen. No significant differences in the effect of both masks were found [9]. 

Wearing masks during the pollen season can be recommended as an effective nonpharmacological option for pollen allergy sufferers, certainly especially on days when high pollen load is predicted. In this way, pollen allergy sufferers would also have some benefit from wearing the mask as additional protection against viruses (e.g. coronaviruses), bacteria or air pollution. 

## Nasal filters 

It is in accordance with an old idea to protect the nose from the entry of pollen by means of a filter. There is some focused research on the effect of nasal filters. 

O’Meara et al. [48] studied the effect of a nasal filter while in a park for 1 hour during the season of grass and ragweed pollen in 48 participants with ARC. It was clear that nasal itching, runny nose, and also sneezing were lower compared to staying in the same place without a nasal filter [48]. 

In 65 subjects with ARC due to grass pollen, it was observed in a DBPC crossover study over 2 days that when wearing a nasal filter (Rhinix, Rhinix ApS, Aarhus, Denmark), the severity in the TNSS, used as outcome parameter, was significantly reduced compared to placebo [36]. Interestingly, fewer tears were present in the eye when wearing the nasal filter. This was also observed when wearing mouth-nose masks [9]. 

The same filter was tested again in a larger group of 1,073 individuals with ARC with or without asthma in the 2014 grass pollen season in Denmark in an open-label study over 2 weeks. The majority of participants were so satisfied with the nasal filter that they wore and appreciated it in their nose over the 2 weeks. Those with more severe asthmatic symptoms and less symptom relief on anti-allergic medication used the filter more than others [37]. 

Wearing nasal filters is certainly not perceived as practical by everyone; its use in practice will therefore probably be limited to individuals who do not feel sufficiently protected by standard medication. 

## Nasal irrigation 

Nasal irrigation is a simple, inexpensive, painless, and relatively common method of preventing nasal symptoms of hay fever. 

In a study of 220 children, who are particularly likely to avoid the use of anti-allergic medications, the positive effect was documented [26]. 

A similar situation exists with pregnant women, who also like to do without any drug therapy. 22 subjects who performed nasal irrigation 3 times daily for 6 weeks during a pollen season had significantly lower nasal symptoms compared to subjects without nasal irrigation [28]. 

Physiological saline solutions as well as hypertonic saline solutions can be used for nasal irrigation; according to one study, the latter have a better effect [45]. The effects of nasal irrigation once or twice daily are felt within the first 4 weeks of starting this practice [27]. 

It is also important to note that nasal irrigation as an adjunct to drug therapy can save ~ 30% of medication while maintaining the same level of symptom control [39]. 

## Pollen forecasts and treatment advice 

Pollen forecasts can be a valuable tool for pollen avoidance as well as management of pollen allergies [38, 57]. They are mainly used during periods of high pollen load [41], demonstrating their value to users. At the same time, a pollen forecast is by no means comparable to a rain forecast, because pollen forecasts lead to important decisions about medication intake for affected individuals. Incorrect predictions can lead to neglect or overdosing of medications with the risk of severe symptoms or dangerous side effects. Therefore, issuing pollen forecasts without medical expertise and careful measurements of actual pollen counts is ethically unacceptable [4, 5]. 

From the abundance of pollen forecasts on the internet, which are intended to provide users with helpful knowledge in managing the disease and in taking preventive medications, very few fulfill these purposes [6]. 

The development of the electronic hay fever diary (PHD = patient’s hay-fever diary) at the Medical School of Vienna [6] was the first evaluated basis for the development of pollen forecasts, which at the same time named the type and severity of allergic symptoms in predicting the risk for the user. This PHD is successfully used by more than 200,000 users/year across Europe in the following years, as it is available in five languages. Analysis of anonymized data provided by users on their nasal, ocular, and bronchial symptoms and medication used led to important findings regarding the definition of pollen season [35] and comparison of symptoms across countries [51]. 

The app “Husteblume”, published in 2015, also uses the PHD and is the only app developed in Germany with a pollen forecast for people with hay fever and pollen-related asthma that has been evaluated for user behavior and effectiveness [29]. Ease of use, impact on quality of life and health literacy, and self-efficacy in managing one’s chronic disease were assessed by two online surveys among 661 registered users of the app before and after the 2017 pollen season. Improvements were seen regarding level of information, quality of life, ability to cope with the disease, and preparation for a doctor’s visit, which, remarkably, was required significantly less frequently (minus 7%). 

A special feature of “Husteblume” is the information on drug therapy, which the user receives within seconds after entering his hay fever-typical symptoms. The user thus can compare his or her own drug therapy with a recommendation [44]. 

It is foreseeable that evaluated and clinically validated apps will be appreciated and used as a matter of course by an increasing number of users in the coming years. Users will present to allergists less frequently after an assured diagnosis and therapy adjustment have been made, which will benefit both patients and allergists. 

## Pollen screens 

Pollen screens are offered on the market, which are supposed to prevent the penetration of pollen into living rooms or bedrooms like a fly or mosquito screen on windows or more extensively in front of doors. Although they have been on the market and in use for years, there are hardly any publications on the subject. 

In one study, the fine fabric from tesa AG (Hamburg, Germany) was tested for its ability to prevent pollen penetration into rooms of an allergy and asthma clinic (Bad Lippspringe, Germany) [7]. 

For this purpose, in two adjacent rooms of the same size (5.2 m wide, 3.4 m long, 2.7 m high) on the third floor of a clinic (11.5 m high) with windows of the same size (132 × 153 cm), parallel measurements of the indoor pollen load were made by two Burkard pollen traps placed on the floor at a distance of 3.0 m from the window. In the “control room” the window was open and covered with or without a fly screen. In the “test room”, pollen screens were stretched in front of the open windows. Measurements were taken daily, and the analysis was performed by an experienced pollen analyst. The rooms were only entered for the operation of the pollen trap from February 7 to July 1, 2002. 

Significant reduction rates (pollen count/24 h behind the pollen screen compared to open window) were obtained: alder: 91.7 – 98.7%, birch: 83.2 – 93.1%, ash: 83.3 – 100.0%, oak: 95.7 – 100%, nettle 89.9 – 100%. Thus, in this study, pollen was effectively prevented from entering the room by the tested pollen screens. 

It would be desirable in further studies to provide evidence that the use of pollen screens not only reduces indoor exposure to pollen, but also reduces clinical symptoms [8]. 

## Nasal ointments, powders, and oils 

The application of ointments, powders, or oils to the nasal mucosa is based on the idea that they act as a barrier to repel pollen absorbed into the nose or prevent the penetration of their allergens into the mucous membranes, thereby preventing inflammatory reactions and symptoms. 

In an open-label, prospective, controlled study, lipid-based nasal ointment suppressed sneezing and itching, but not runny nose and nasal mucosal swelling, compared with untreated subjects [30]. A DBPC study in ~ 100 subjects showed a reduction of ~ 60% in nasal symptoms in response to nasal provocation by the pollen-blocking nasal cream as compared to placebo, which resulted in a reduction of only 25% [59]. 

A large DBPC study in patients with ARC due to birch, grass, or olive pollen showed good tolerability of the nasal emulsion but little effect on organ-specific quality of life, and only the symptom of nasal congestion was significantly reduced [47]. 

In the UK, cellulose powder has been on the market as an anti-hay fever agent since 1994 and Emberlin and Lewis published the first controlled study in 2006 [22]. The efficacy of the powder was tested in comparison with a placebo in 97 adults with ARC due to grass pollen with the question of who needed more additional medication during the pollen season. The cellulose powder was convincing: the 47 adults under cellulose needed significantly less additional anti-allergic drugs. However, applying cellulose powder to the nasal mucosa alone did not reduce symptoms by itself but only in conjunction with less medication; this may also be considered a success, but is not the real purpose of an intervention. 

In a DBPC study, Aberg et al. [1] were able to significantly reduce residual nasal, ocular, and bronchial symptoms in 53 children and adolescents with ARC due to birch pollen, all of whom were on oral anti-allergic medication (anti-histamine), when cellulose powder was additionally applied – preferably on days with relatively low concentrations of airborne birch pollen (≤ 100/m^3^). 

In another controlled study by Aberg et al. [2] (same design), 108 adults with confirmed grass pollen rhinitis also responded with significantly less sneezing, runny nose, nasal congestion, and eye and bronchial symptoms; the experienced allergists then recommended the use of cellulose powder at the onset of initial symptoms in the pollen season. 

In 2017, Popov et al. [55] summarized all available data on the use of hydroxy-propyl-methyl-cellulose powder (HPMC-p) from the 26 studies published using HPMC-p. As a result, they were able to convincingly conclude that the now patented method of using HPMC is an effective barrier against penetrating airborne allergens, including pollen, and is capable of reducing nasal symptoms. 

Popov et al. [56] additionally tested the effect of HPMC in the nose as an effective barrier to allergen extracts from both pollen of cedar, ragweed, and grasses and from house dust mites and particulate matter (PM_2.5_) using animal experiments (rats). The HPMC-p itself resulted in no inflammatory tissue reactions for up to 48 hours after application and was able to significantly reduce allergen extracts and PM_2.5_ as they entered the tissue. 

In a non-reviewed preprint communication literature site [46], the efficacy of hydroxypropyl methylcellulose (Nasaleze International Ltd., Isle of Man, British Isles, UK) was tested in 36 adults with AR due to grass pollen (*Dactylis glomerata*) in the Fraunhofer Allergen Exposure Chamber Hannover in an open-label cross-over study. TNSS, nasal secretion, and symptoms were significantly reduced by HPMC for at least 4 hours. 

Overall, the large number of studies shows that cellulose powder in the nose is an effective barrier against the penetration of allergens and airborne particulate matter. 

## Sunglasses and contact lenses 

In a study of 70 adults [20], wearing sunglasses with the rim pulled around (wraparound glasses) in addition to medication (group 1, n = 39) or without medication (group 2 = 31) was assessed over pollen periods in 3 years using symptoms, medication needed, and the RQLQ. Eye itching and redness, sneezing, and runny nose were significantly reduced by the sunglasses, while quality of life was improved. 

The protective effect of sunglasses regarding ocular symptoms in 39 subjects with confirmed ARC due to pollen and excluded sensitization to perennial allergens was performed in two groups: group 1 received topical steroids regularly and loratadine as needed; group 2 wore the sunglasses in addition to the same medication. Sunglasses significantly reduced ocular symptoms (p = 0.002) and the need for antihistamines (p = 0.009) [49]. 

No publications could be found on the possible protective effect of contact lenses. 

## Conclusion and outlook 

According to the current knowledge, individual interventions, such as pollen filters in cars, can be recommended, but only as an add-on therapy in combination with standard drug therapy. Here it is important to identify and address concerns during the patient interview, as fortunately with modern antihistamines and nasal steroids, effective and well tolerated therapies are available even in long-term treatment. Thus, no cumulative or long-term side effect has ever been described for antihistamines despite their use in hundreds of millions of patients treated. 

Allergen immunotherapy is the only available disease-modifying therapeutic option in the treatment of patients with type-mediated allergy. A high body of scientific evidence is available demonstrating the efficacy and safety of this form of therapy. 

The extraordinary frequency of ARC due to pollen in its various degrees of severity and accompanying symptoms (outside the eyes, nose, and bronchial tubes) on the skin, in the throat, and in the general condition leads to the fact that also untested non-drug procedures are tried. The very individual experiences and observations with such untested procedures can lead to the recommendation of further methods, not listed here, in the community of affected persons or in the public. As an example, the frequently mentioned hair washing; here, the lay press simply copies from each other again and again without questioning the value. 

Data-based information for the millions of sufferers of ARC remains the most important viable basis for the use of non-medicinal measures. The German Pollen Information Service Foundation is the most important source for this (www.pollenstiftung.de). 

## Funding 

The manuscript was produced without financial help from a third party. 

## Conflict of interest 

K.C.B. is Chairman of the Board of the German Pollen Information Service Foundation (PID). 

B.W .is a member of the Board of Directors of the PID. BW and MW are research fellows of the PID. 

O.P. reports grants and personal fees from ALK-Abelló, grants and personal fees from Allergopharma, grants and personal fees from Stallergenes Greer, grants and personal fees from HAL Allergy Holding B.V./HAL Allergie GmbH, grants and personal fees from Bencard Allergie GmbH/Allergy Therapeutics, grants and personal fees from Lofarma, grants from Biomay, grants from Circassia, grants and personal fees from ASIT Biotech Tools S.A., grants and personal fees from Laboratorios LETI/LETI Pharma, personal fees from MEDA Pharma/MYLAN, grants and personal fees from Anergis S.A., personal fees from Mobile Chamber Experts (a GA2LEN Partner), personal fees from Indoor Biotechnologies, grants and personal fees from GlaxoSmithKline, personal fees from Astellas Pharma Global, personal fees from EUFOREA, personal fees from ROXALL Medizin, personal fees from Novartis, personal fees from Sanofi-Aventis and Sanofi-Genzyme, personal fees from Med Update Europe GmbH, personal fees from streamedup! GmbH, grants from Pohl-Boskamp, grants from Inmunotek S.L., personal fees from John Wiley and Sons, AS, personal fees from Paul-Martini-Stiftung (PMS), personal fees from Regeneron Pharmaceuticals Inc., personal fees from RG Aerztefortbildung, personal fees from Institut für Disease Management, personal fees from Springer GmbH, personal fees from AstraZeneca, personal fees from IQVIA Commercial, personal fees from Ingress Health, outside the submitted work; and member of the Executive Committee (ExCom) of the European Academy of Allergy and Clinical Immunology (EAACI) and of the (ext.) board of directors of the German Society of Allergy and Cliical Immunology (DGAKI). Coordinator and/or member of many guidelines and Position Papers in the field of allergic diseases. 

M.B., L.K. and T.Z. declare no conflict of interest with the content of the manuscript. 

**Table 1.** Overview of methods and estimation of risk of use, effort/cost, frequency of communication or experience, and a recommendation for use.

Method	Risk	Effort/Cost	Frequency/Experience	Recommendation
Acupuncture	1	3	3	1
Car filter	0	2	3	3
Endonasal phototherapy	3	3	1	0
Hypnosis	1	3	1	1
Washing clothes	0	1	1	2
Artificial tears	1	2	1	1
Air purifiers	0	3	3	3
Masks	0	1	2	3
Nasal filters	0	2	2	2
Nasal irrigation	0	0	2	3
Pollen forecasts	0	1	3	3
Pollen screens	0	2	2	3
Nasal ointments etc.	1	2	2	1
Sunglasses	0	2	2	3

Score: rating: 0 = none, 1 = low, 2 = medium, 3 = high. The data reflect the authors’ assessment and are not evidence-based because the methods are too different to compare against each other.
